# Suppressive Effects of Octyl Gallate on *Streptococcus mutans* Biofilm Formation, Acidogenicity, and Gene Expression

**DOI:** 10.3390/molecules24173170

**Published:** 2019-08-31

**Authors:** Vika Gabe, Tomas Kacergius, Saleh Abu-Lafi, Mouhammad Zeidan, Basheer Abu-Farich, Donatas Austys, Mahmud Masalha, Anwar Rayan

**Affiliations:** 1 Department of Physiology, Biochemistry, Microbiology and Laboratory Medicine, Institute of Biomedical Sciences, Faculty of Medicine, Vilnius University, Vilnius 03101, Lithuania; 2 Faculty of Pharmacy, Al-Quds University, Abu-Dies 144, Palestine; 3 Molecular Genetics and Virology Laboratory, QRC-Qasemi Research Center, Al-Qasemi Academic College, P.O. Box 124, Baka EL-Garbiah 30100, Israel; 4 Science Education Department, Al-Qasemi Academic College, P.O. Box 124, Baka EL-Garbiah 30100, Israel; 5 Department of Public Health, Institute of Health Sciences, Faculty of Medicine, Vilnius University, Vilnius 03101, Lithuania; 6 Microbiology Laboratory, QRC-Qasemi Research Center, Al-Qasemi Academic College, P.O. Box 124, Baka EL-Garbiah 30100, Israel; 7 The Institute of Applied Research - Galilee Society, P.O. Box 437, Shefa-Amr 20200, Israel; 8 Faculty of Science, Al-Qasemi Academic College, Baka El-Garbiah 30100, Israel

**Keywords:** octyl gallate, *Streptococcus mutans*, biofilm, acidogenicity, gene expression

## Abstract

The accumulation of biofilm by *Streptococcus mutans* bacteria on hard tooth tissues leads to dental caries, which remains one of the most prevalent oral diseases. Hence, the development of new antibiofilm agents is of critical importance. The current study reports the results from testing the effectiveness of octyl gallate (C8-OG) against: (1) *S. mutans* biofilm formation on solid surfaces (polystyrene, glass), (2) acidogenicity, (3) and the expression of biofilm-related genes. The amount of biofilm formed by *S. mutans* bacteria was evaluated using the colorimetric method and optical profilometry. The pH of the biofilm growth medium was measured with microelectrode. A quantitative reverse transcription-polymerase chain reaction (RT-qPCR) was used to assess the expression of genes encoding glucan binding protein B (*gbpB*), glucosyltransferases B, -C, -D (*gtfB, -C, -D*), and the F-ATPase β subunit of the F_1_ protein (*atpD*). The results show that C8-OG significantly diminished biofilm formation by exposed *S. mutans* on solid surfaces and suppressed acidogenicity in a dose-dependent manner, compared to unexposed bacteria (*p* < 0.05). The C8-OG concentration of 100.24 µM inhibited *S. mutans* biofilm development on solid surfaces by 100% and prevented a decrease in pH levels by 99%. In addition, the RT-qPCR data demonstrate that the biofilm-producing bacteria treated with C8-OG underwent a significant reduction in gene expression in the case of the four genes under study (*gbpB*, *gtfC*, *gtfD*, and *atpD*), and there was a slight decrease in expression of the *gtfB* gene. However, C8-OG treatments did not produce significant expression change compared to the control for the planktonic cells, although there was a significant increase for the *atpD* gene. Therefore, C8-OG might be a potent antibiofilm and/or anticaries agent for oral formulations that aim to reduce the prevalence of dental caries.

## 1. Introduction

Dental biofilm formation is primarily induced by *Streptococcus mutans* bacteria colonizing the human mouth [1]. The development of biofilm on tooth surfaces results from the cooperative action of the *S. mutans* glucosyltransferases, GtfB, -C, and -D, which use dietary sucrose to synthesize water-insoluble, partly soluble, and soluble glucan polymers, respectively [2]. Moreover, the *S. mutans* glucan-binding proteins GbpA, -B, -C, and -D, assist with the adhesion of the bacteria to glucans, thereby enabling the biofilm to mature [3]. The subsequent bacterial fermentation of carbohydrates (e.g., sucrose) into organic acids causes the acidification of dental biofilm [1]. The acidogenicity within *S. mutans* biofilm is further maintained by bacterial proton extruding F-type ATPase composed of the membrane-embedded F_0_ protein and peripherally bound F_1_ protein complexes, with their most important b and β subunits encoded by *atpF* and *atpD* genes, respectively [4]. Consequently, the constant acidogenicity of the biofilm induces tooth demineralization, leading to cavitation or dental caries, which continues to exhibit high global prevalence, despite the availability of various prophylactic measures [5,6]. Thus, there is a need for the development of new antibiofilm and/or anticaries agents.

In recent years, further active phytochemicals derived from natural sources have been intensively investigated for their action against bacterial pathogens as a result of the emergence of multiple drug resistance [7,8,9,10,11,12]. In a preceding study, we investigated the effects of ethyl gallate (C2-EG) on genes involved in biofilm production by *S. mutans.* [13] Moreover, the inhibitory capacity of methyl gallate (C1-MG) on *S. mutans* biofilm formation by optical profilometry has been evaluated [14]. The current work, however, aimed to explore the effect of octyl gallate (C8-OG)—a compound like C2-EG, but with a longer alkyl chain—on biofilm formation, using an optical profilometry assay, and to identify the genes associated with this bioactivity.

Gallic acid (GA) and C8-OG were isolated and identified in *Terminalia bellerica* methanolic extract [15,16]. *T. bellirica* fruits inhibited the growth of drug-resistant wild strains of *S. aureus, Acinetobacter* spp., *P. aeruginosa*, and *E. coli.* [17] It has also been reported that *T. bellirica* extract exhibited pronounced antioxidant activity and moderate anti-apoptotic and hepatoprotective potential [18]. Octyl gallate and gallic acid isolated from *T. bellerica* regulate the normal cell cycle in human breast cancer cell lines [16]. Gentamicin and trimethoprim, in combination with gallotannin 1,2,6-tri-*O*-galloyl-β-d-glucopyranose, a compound isolated from *Terminalia chebula* fruits, has been reported to possess the potential to treat urinary tract infections caused by biofilm-forming, multidrug-resistant, uropathogenic *E. coli.* [19]. Moreover, in vitro biofilm-inhibiting activity by C1-MG, isolated from *Terminalia chebula* against fluoroquinolone-resistant *Vibrio cholera*, has been reported [20]. C8-OG has shown significant antiviral, antifungal, and antibacterial potential [21,22,23] and has been used as an antioxidant and preservative [24,25]. The current study reports the results from testing the effectiveness of C8-OG against: (1) *S. mutans* biofilm formation on solid surfaces (polystyrene, glass), (2) acidogenicity, (3) and the expression of biofilm-related genes.

## 2. Results and Discussion

**Figure 1:**
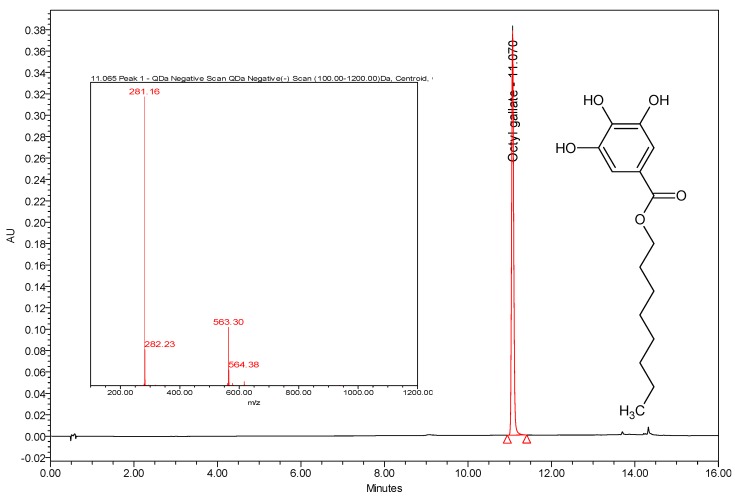
Typical HPLC-PDA chromatogram of 250 ppm of C8-OG standard at its maximum wavelength of 272 nm, with its chemical structure and MS.

**Figure 2:**
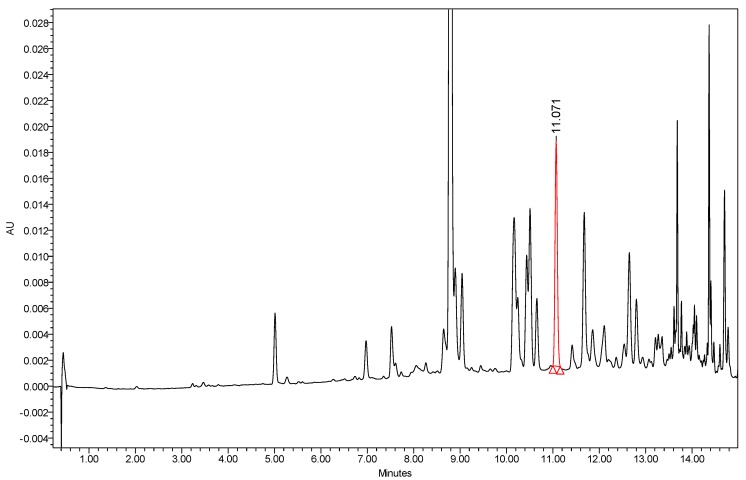
Zoomed view of the HPLC-PDA chromatogram of *Terminalia bellerica* extract from hexane; base-line separation of the C8-OG peak is seen in red, eluted at 11.071 min.

Octyl gallate (C8-OG) is the ester of the surfactant-like 1-octanol and the hydrophilic gallic acid. It is naturally present in the fruits of *Terminalia bellerica* [15,16]. Four freshly prepared extracts of water, ethanol, ethyl acetate, and hexane were isolated from *Terminalia bellerica* and injected directly into the LC-PDA-MS machine. A typical LC-PDA chromatogram of standard C8-OG, along with its chemical structure and MS, is shown in Figure 1. C8-OG presents in all four extracts, but in smaller amounts in the aqueous and ethanolic extracts compared to the hexane and ethyl acetate extracts (0.19% wt/wt in hexane, 0.11% in ethyl acetate, 0.027% in water, and 0.018% in ethanol). The retention time of the well resolved C8-OG peak in the hexane extract is shown in Figure 2. Using the negative ESi mode, a typical pseudo 11-peak, at a mass-to-charge ratio of 281 Da, can be seen (Figure 1). An adduct dimer of [2M − 1]^−^ is also seen at *m/z* of 563 Da, apparently because of the relatively large concentration of C8-OG injected standard.

### 2.1. Antibacterial Activity of C8-OG on S. mutans

As shown in Table 1, the value of the minimum inhibitory concentration (MIC) of C8-OG on *S. mutans* is 97 µg/mL (343.5 µM).

### 2.2. Suppressive Activity of C8-OG on S. mutans Biofilm Formation on Polystyrene Surfaces

**Figure 3:**
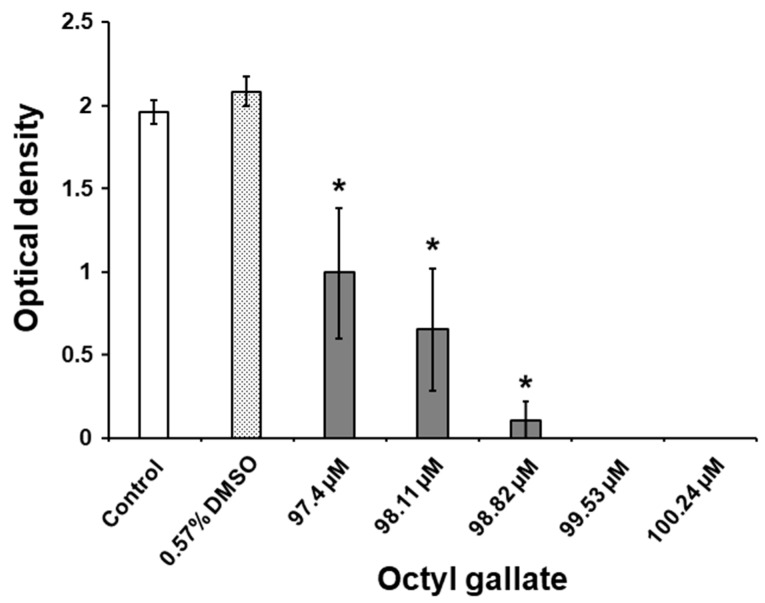
Effects of C8-OG on the biofilm biomass formed by *S. mutans* on polystyrene surfaces after 24 h of incubation in Todd Hewitt broth (THB) containing 1% sucrose. The data represent the mean ± standard error from three separate experiments (*n* = 3−9). * *p* < 0.05 versus the control group.

As shown in Figure 3, the biofilm-inhibiting C8-OG concentrations of 97.4 µM, 98.11 µM, 98.82 µM, 99.53 µM, and 100.24 µM fall into a very narrow range, but are lower than the determined MIC (i.e., 343.57 µM). This indicates that this inhibitory activity was not related to the bactericidal effect of C8-OG. The colorimetric method revealed that all the tested concentrations of C8-OG significantly decreased *S. mutans* biofilm biomass on the polystyrene surfaces, compared to the biomass of untreated bacteria (*p* < 0.05) growing in Todd Hewitt broth (THB) with 1% sucrose (Figure 3). This suppressive activity of C8-OG on biofilm formation occurred in a dose-dependent manner. Importantly, the C8-OG concentrations of 99.53 µM and 100.24 µM inhibited *S. mutans* biofilm development on the polystyrene surfaces by 100%, versus the untreated bacteria (Figure 3). Furthermore, the exposure of bacterial cells to DMSO alone at a concentration of 0.57% did not significantly affect biofilm formation on the polystyrene surfaces in comparison to the control bacteria (*p* > 0.05). It is important to note that the inhibitory activity of C8-OG on *S. mutans* biofilm formation on the polystyrene surfaces was considerably higher than the like effect of ethyl and methyl gallates, as reported by Gabe et al. [13] and Kacergius et al. [14] In this regard, the concentration ranges were 2.78–3.53 mM and 2.99–5.43 mM for ethyl and methyl gallates, respectively. This finding could be reasonably related to the longer alkyl chain in the chemical structure of octyl gallate, which might explain its better antibiofilm activity. In addition, a recent investigation by Oh et al. [26] demonstrated a similar suppressive effect by octyl gallate on the production of biofilm biomass by another gram-positive bacterium—*Staphylococcus aureus*. 

### 2.3. Suppressive Activity of C8-OG on S. mutans Biofilm Formation on the Glass Surfaces

**Figure 4:**
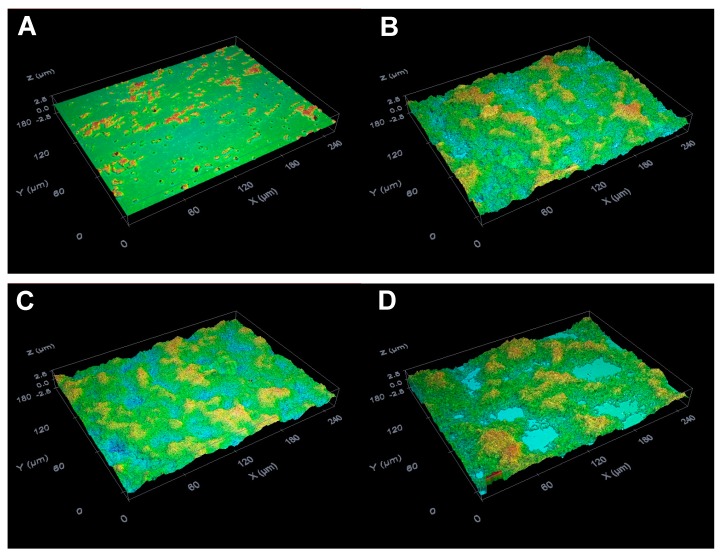

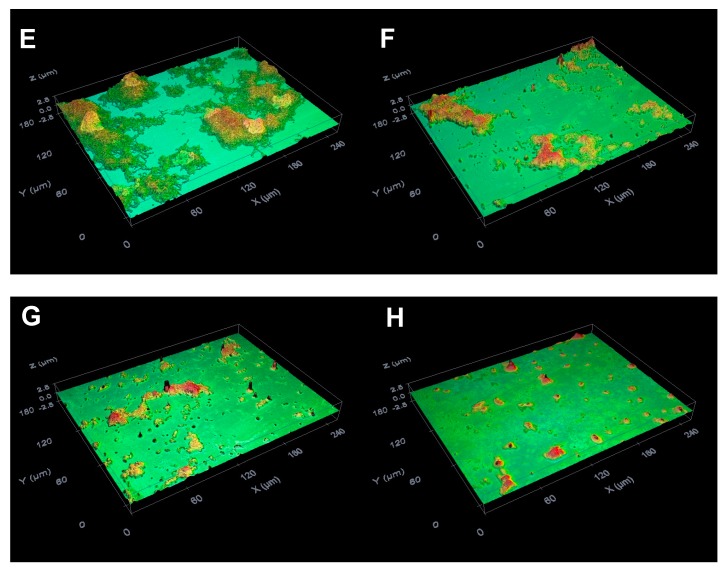
Effects of C8-OG on *S. mutans* biofilm formation on the glass surfaces after 24 h of incubation. Optical profile of the glass slide surfaces with bacteria incubated (**A**) without C8-OG and sucrose, (**B**) without C8-OG, in the presence of 1% sucrose, and (**C**) exposed to 0.57% DMSO or C8-OG at concentrations of (**D**) 97.4 µM, (**E**) 98.11 µM, (**F**) 98.82 µM, (**G**) 99.53 µM, and (**H**) 100.24 µM. Magnification, ×50.

**Figure 5:**
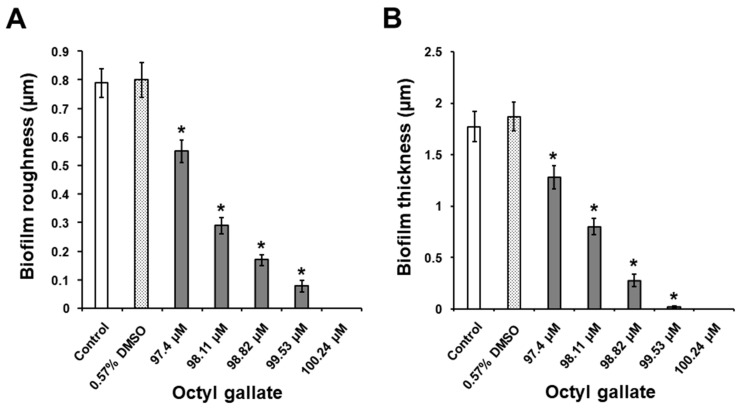
Effects of C8-OG on the quantities of biofilm formed by *S. mutans* on the glass surfaces after 24 h of incubation in THB containing 1% sucrose. (**A**) The surface roughness parameter (*R*_q_) of the biofilm on the glass slides and (**B**) the biofilm thickness. The data represent the mean ± standard error from three separate experiments (*n* = 18, biofilm roughness; *n* = 15, biofilm thickness). * *p* < 0.05 versus the control group.

Analysis of the surfaces of the glass slides using the optical profilometry technique revealed that the presence of 1% sucrose in THB considerably stimulated *S. mutans* biofilm development on the glass surfaces (Figure 4B and Figure 5) in comparison to the bacteria grown without C8-OG and sucrose (Figure 4A). For the latter bacteria, the *R*_q_ and thickness parameters were 0.1 ± 0.01 and 0.16 ± 0.01 μm, respectively. In addition, it should be noted that the exposure of bacteria to DMSO alone at a concentration of 0.57% did not significantly affect biofilm formation, (Figure 4B,C, and Figure 5). However, with the THB containing 1% sucrose, the treatment of bacteria with C8-OG suppressed *S. mutans* biofilm formation on the glass surfaces in a dose-dependent manner (Figure 4D–H, and Figure 5). C8-OG concentrations of 97.4, 98.11, 98.82, 99.53, and 100.24 µM significantly reduced the surface roughness parameter (*R*_q_) of the biofilm (Figure 5A) and the biofilm thickness (Figure 5B), compared to those of the control bacteria (*p* < 0.05). It is important to emphasize that the C8-OG concentration of 100.24 µM was able to completely inhibit (i.e., by 100%) *S. mutans* biofilm development on the glass surfaces. In contrast to our previous results obtained using ethyl and methyl gallates [13,14], substantially lower concentrations of octyl gallate were needed to suppress *S. mutans* biofilm build-up on the glass surfaces, which points to the importance of the increased alkyl chain for obtaining a greater antibiofilm effect.

### 2.4. Suppressive Activity of C8-OG on S. mutans Biofilm Acidogenicity

Evaluation of the pH levels in the *S. mutans* biofilm growth medium showed considerable acidification of the biofilm grown without C8-OG in the presence of 1% sucrose. In this respect, the fermentation of sucrose by the bacteria in the control group induced an ~1.7-fold lowering of the pH, versus the pH of the blank group (Table 2). Exposure of the bacteria to DMSO alone at a concentration of 0.57% did not affect the acidification of *S. mutans* biofilm, judging from the pH levels presented in Table 2. However, the production of acids was significantly inhibited by the treatment of *S. mutans* bacteria with C8-OG, in comparison to the untreated control bacteria (*p* < 0.05), by raising the pH approximately to the pH levels of the blank group (Table 2). The C8-OG increased the pH levels in a dose-dependent manner. It is important to note that C8-OG concentrations from 98.82 µM to 100.24 µM prevented a decrease in the pH by 98%–99%. Thus, octyl gallate possesses the potential to suppress the acidogenicity of *S. mutans* biofilm. It is worth noting that this inhibitory activity was attained by applying considerably lower concentrations of octyl gallate than the concentrations of ethyl and methyl gallates used in our previous studies [13,14].

### 2.5. Gene Expression Analysis

**Figure 6:**
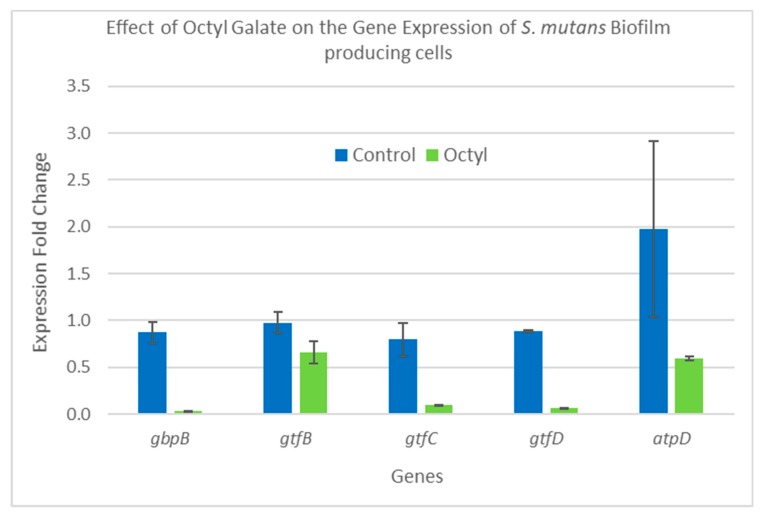
The effects of C8-OG treatment on the expression of five *S. mutans* genes involved in biofilm production. *S. mutans* cells were collected from the biofilm phase. C8-OG means treatment with C8-OG at a 24.25 µg/mL concentration (comparable to 1/4 of the minimum inhibitory concentration (MIC) value), shown in green, while the bars in blue represent the untreated controls.

**Figure 7:**
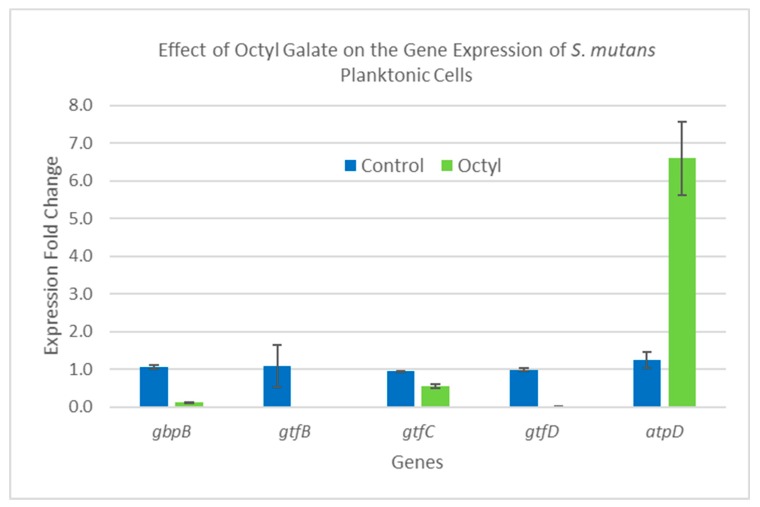
C8-OG effects on the expression of six *S. mutans* genes that are involved in biofilm production. The *S. mutans* cells were collected from planktonic growth. C8-OG means treatment with octyl gallate at a 24.25 µg/mL concentration (comparable to 1/4 of the MIC value), shown in green, while the bars in blue represent the untreated controls.

SYBR qRT-PCR was utilized to determine the relative change in expression of five biofilm-related genes—*gbpB*, *gtfB*, *gtfC*, *gtfD*, and *atpD*, as well as a reference gene termed *16SrRNA.* Seeded *S. mutans* cells producing biofilm and planktonic cells were treated with three concentrations of C8-OG (97 µg/mL, 48.5 µg/mL, and 24.25 µg/mL), as well as a control of untreated cells. Since the OG 1.0 and 0.5 MIC samples showed no growth, or a negligible amount of *S. mutans*, only the 0.25 MIC sample was used for the gene expression analysis. The results of the gene expression analysis using the two steps of the qRT-PCR are shown as gene expression fold changes in the bars of the graph. Figure 6 summarizes the results for the biofilm-producing cells, and Figure 7 for the planktonic cells. In Figure 6, the biofilm-producing cells treated with C8-OG at a concentration of 24.25 µg/mL (labeled OG and equal to 25% of the MIC value) show a significant reduction in four of the five genes tested. For instance, high reduction in gene expression was obtained for *gbpB* (a 98.6 increase in fold change), *gtfC* (a 47.5 increase in fold change), *gtfD* (a 13.8 increase in fold change), and *atpD*, and there was a slight non-significant decrease in the *gtfB* gene. These results are highly in agreement with the colorimetric, profilometric, and acidogenicity results. The effect of C8-OG on the downregulation of all genes could explain the effect on glucan synthesis and matrix rigidity, biofilm production, and the damage to the biofilm. It also indicates that disruption of the cooperative action of all three glucosyltransferases led to the inhibition of sucrose-dependent *S. mutans* biofilm formation. These results show that the addition of octyl residues to gallate assisted in penetrating the biofilm matrix, as well as reaching the embedded glucan-producing bacteria, and produced the salient effect. In this context, this could explain why the glucan synthesis encoding genes were not inhibited by ethyl gallate, as reported in our recent study [10]. In contrast, Figure 6 shows that C8-OG treatment of the planktonic cells caused a slight and non-significant decrease in four genes (*gbpB*, *gtfB*, *gtfC*, and *gtfD*) and a significant up-regulation for the expression of the *atpD* gene only. Altogether, our results show that the biofilm and planktonic cells were affected differently by the C8-OG treatment. However, the results show high agreement with the colorimetric, profilometric, and pH measurements. Another explanation for these results could be that the inhibition of biofilm formation on polystyrene and glass surfaces, and of acidogenicity, by octyl gallate occurs at the level of gene expression of the five genes, which are involved in the pathway of glucan synthesis and binding.

More studies will be conducted in the future to investigate the effects of different gallate derivatives on the same genes used in this study, which are known to be involved in biofilm production and maintenance on the genetic level.

## 3. Materials and Methods

### 3.1. The Source of Chemicals

Acetonitrile (ACN) LCMS-grade solvent was purchased from Merck (Darmstadt, Germany). Highly purified water was prepared with a Millipore Milli-Q Plus water purification system. Octyl gallate, analytical standard, was purchased from Sigma, Rehovot, Israel.

### 3.2. Terminalia Bellerica Plant Extraction

To extract the octyl gallate, *Terminalia bellerica* fruits were ground up with a grinding machine; packed in four tubes, each containing one gram of plant material soaked in 20 milliliters of solvent (water, ethanol, ethyl acetate, and hexane); sonicated for 90 min at 40 °C; and then left for 3 h to cool down. After extraction was completed, the extract solutions were filtered with Whatman paper, grade 1, followed filtering at 0.2 µm. Ten milliliters from each solution were dried in a vacuum to determine the concentration, while the rest were kept at 4 °C until they were used.

### 3.3. Instrumentation and Chromatographic Conditions

The analyses were performed on a Waters Acquity UPLC H-Class system (Waters, Milford, MA, USA) equipped with a binary solvent manager, sampler manager, column manager, and an Acquity QDa detector and PDA connected to a Waters Empower 3 data station. An Acquity UPLC BEH C18 column (50 mm × 2.1 mm I.D., 1.7 μm) equipped with an Acquity BEH C18 1.7 μm guard column (Vanguard 2.1 mm × 5 mm) was also from Waters, Milford, MA, USA. All the samples were filtered with a 0.45-µm PTFE filter. The PDA wavelengths ranged from 210 nm to 500 nm. The flow rate was 0.4 mL/min. The injection volume was 1 µL, and the column temperature was set at 25 °C. The LC-MS column and sample temperature were maintained at 40 °C and 25 °C, respectively. The mobile phase consisted of 0.1% formic acid in water (A) and 0.1% formic acid in acetonitrile (B) at a constant flow rate of 0.4 mL/min. The initial linear gradient starting conditions were 98% A for 1 min, increased to 100% B over 12 min. Then it was held at 100% B for an extra two minutes. Prior to any injection, the mobile phase was equilibrated for 4 min. The MS ionization modes were in the negative and positive ESi, the mass range was between 100 and 1200 Da, the cone voltage was 15 V, the capillary temperature was 600 °C, and the capillary voltage was 0.8 KV, and a sampling rate of 8 points/second was used.

### 3.4. Calibration Curve of Octyl Gallate

C8-OG standard injections at concentration levels of 10, 50, 100, and 250 ppm generate a linear line with an R^2^ value of 0.9995. A linear calibration curve was used to determine the weight-by-weight percentage (% wt/wt) concentration of C8-OG in the four extracted samples of *Terminalia bellerica*.

### 3.5. Bacterial Culture

*Streptococcus mutans* strain UA159 (ATCC No. 700610) was purchased from the American Type Culture Collection (Manassas, VA, USA). The bacterial culture was stored in 10% skim milk (Difco; BD BioSciences, Franklin Lakes, NJ, USA) at −70 °C until use. Before beginning of the experiments, *S. mutans* was cultured in Bacto^™^ Todd Hewitt broth (THB; BD BioSciences, NJ, USA) using anaerobic conditions (95% N_2_ and 5% CO_2_) at 37 °C for 18 h. The purity of the cultures was assessed on Mitis Salivarius agar (Difco; BD BioSciences, NJ, USA) and Columbia agar, with 7% sheep blood (E&O Laboratories, Bonnybridge, Scotland).

### 3.6. Microdilution Test for Determining the Minimum Inhibitory Concentration (MIC)

To determine the MIC value, the broth microdilution assay was applied using a two-fold serial dilution of C8-OG solution in Brain heart infusion (BHI) broth, as described elsewhere [13]. Each well contained 10^5^ clone-forming units. The MIC value was defined as the lowest concentration that can inhibit the visible growth of bacteria in triplicate wells, following incubation at 37 °C for 24 h overnight. After the MIC value was visually determined, 20 µL of *p*-iodonitrotetrazolium violet (8 mg/mL in ethanol) were added to each well. The plate was incubated for another 30 min and inspected visually for any change in color from yellow to pink, which indicates a chemical reduction of dye due to bacterial growth. A two-fold dilution of erythromycin was used as a positive control.

### 3.7. Protocol for the Biofilm Development and Treatment with C8-OG

To determine the antibiofilm activity of C8-OG, the development of *S. mutans* biofilm on polystyrene and glass surfaces was investigated in distinct experiments. Before beginning each experiment, the optical density (OD) of the *S. mutans* culture was adjusted to 0.2 at 630 nm in order to obtain 1.6 × 10^8^ bacterial cells/mL, using a microplate-reader spectrophotometer. To develop *S. mutans* biofilm on the polystyrene surfaces, 24-well, flat-bottomed, polystyrene cell culture plates (Sarstedt, Nümbrecht, Germany) were filled with THB containing 1% sucrose, and then a solution of octyl gallate (i.e., C8-OG; Sigma-Aldrich, Merck KGaA, Darmstadt, Germany), prepared in pure dimethyl sulfoxide (DMSO; Sigma-Aldrich, Merck KGaA, Darmstadt, Germany), was added to the appropriate wells at final concentrations of 27.5 µg/mL (97.4 µM), 27.7 µg/mL (98.11 µM), 27.9 µg/mL (98.82 µM), 28.1 µg/mL (99.53 µM), and 28.3 µg/mL (100.24 µM). Moreover, DMSO, the solvent of C8-OG, was added to the appropriate wells at the highest concentration used in the experiments, 0.57% (v/v). To develop *S. mutans* biofilm on the glass surfaces, sterile glass slides 1-mm thick, cut from standard microscope slides (76 mm × 26 mm; Thermo Fisher Scientific, Inc., Waltham, MA, USA), were put vertically into the plate wells. Afterwards, the bacterial cells were introduced into the wells at a final dilution of 1:100, and all of the plates were incubated under anaerobic conditions (95% N_2_ and 5% CO_2_) at 37 °C for 24 h. The biofilm that formed on the polystyrene and glass surfaces was then quantified by applying colorimetric and optical profilometry methods, respectively. In these experiments, plate wells without bacterial cells were used as blank controls, and untreated bacteria served as experimental controls. In the colorimetric and optical profilometry procedures, the percentage of the inhibition of biofilm development was calculated using the values of OD, surface roughness (*R*_q_), and biofilm thickness parameters, with the following formula:

(1) $$\%\;\mathrm{of}\;\mathrm{the}\;\mathrm{inhibition}=\left(\frac{{\mathrm{Parameter}}_{untreated\;control}\;-{\;\mathrm{Parameter}}_{treatment}}{{\mathrm{Parameter}}_{untreated\;control}}\right)\times 100\%.$$

### 3.8. Biofilm Analysis by the Colorimetric Method

*S. mutans* biofilm development on the polystyrene surfaces was assessed by quantifying the biofilm biomass using the colorimetric method, as described previously [13,14]. Briefly, the fixed and air-dried biofilm in the plate wells was stained with a 0.01% crystal violet solution (Merck KGaA, Darmstadt, Germany), and then the bound dye was extracted using a 33% acetic acid solution (Merck KGaA, Darmstadt, Germany). Afterwards, the OD of the extracted dye solution of the samples was measured at a wavelength of 595 nm with a microplate-reader spectrophotometer. Background staining was corrected for by subtracting the amount of the staining in the blank wells and in the wells with untreated bacteria grown in the absence of sucrose.

### 3.9. Biofilm Analysis by the Optical Profilometry Method

*S. mutans* biofilm formation on the glass surfaces was evaluated by the optical profilometry method, following the procedures outlined in our previous studies [13,14]. In brief, the air-dried glass slides with adherent *S. mutans* biofilm were analyzed using a non-contact optical imaging profilometer Sensofar PLµ 2300 system (Terrassa, Spain) and by applying a 50× confocal objective with a view field of 253 μm × 190 μm. At first, six regions, and then five regions, of the glass slide were scanned in a vertical mode, halfway from the bottom to the top of the visible biofilm, to evaluate the surface roughness and the biofilm thickness, respectively. The data collected from the images were further processed with Gwyddion software (version 2.50, Department of Nanometrology, Czech Metrology Institute, Brno, Czech Republic; http://gwyddion.net) to calculate the parameters for surface roughness and biofilm thickness. The root mean square for the roughness (*R*_q_) parameter was calculated to quantitatively assess the slide surface roughness as an indication of the adherence of bacteria. The height of an artificially produced vertical scratch on each slide with adherent bacteria was used to measure and calculate the biofilm thickness, which specified the maturity of the biofilm. The background for the parameters of surface roughness and biofilm thickness was corrected for by subtracting the *R*_q_ and thickness values of the blank glass slides, and those of the glass slides with untreated bacteria grown in the absence of sucrose.

### 3.10. Evaluation of the Biofilm Acidogenicity

The acidification of *S. mutans* biofilm was determined on the basis of a previously described protocol [13]. The pH of the biofilm growth medium was measured with a microelectrode InLab^®^ Micro Pro ISM^®^ connected to a bench-top pH meter, a SevenCompact^™^ S210-Bio (Mettler-Toledo GmbH, Greifensee, Switzerland), and was calibrated using standard pH buffers (pH 4.01 and 7.00).

### 3.11. Analysis of Gene Expression

Overnight cultures of *S. mutans* grown in LB broth were diluted into a fresh LB medium of 1% sucrose to obtain a final concentration of 0.5 × 10^5^ clone-forming units per milliliter (CFU/mL) and were equally distributed into 50-mL tubes (30 mL/tube). Different concentrations of octyl gallate were added to each tube to reach final concentrations of 343.5 µM, 171.75 µM, and 85.88 µM. Cultures without octyl gallate were used as a control. Cells were grown in three wells of 6-well plate at 5 mL per well (15 mL in total), and the plates were incubated at 37 °C for 24 h planktonic cells were collected separately and stored at −4 °C for further analysis. The attached cells (biofilm) were scraped from the wells and stored at −4 °C for RNA isolation.

### 3.12. RNA Isolation

After harvesting the bacteria by centrifuging the cultures at 4800× *g* for 10 min, the bacterial pellet was suspended in 500 µL of ice-cold phosphate buffer solution (PBS). The suspension was then centrifuged at 500× *g* for 10 min at 4 °C. This washing step was repeated twice. The RNA was extracted by a combination of the CTAB and QIAGEN RNeasy Mini Kit methods. In short, CTAB/PVP/BME solution was added to the bacteria pellet and, after resuspension, subjected to three cycles of deep freezing at −80 °C, followed by thawing at room temperature to allow the lysing of the cells, and was subsequently processed as described by Jordon-Thaden et al. [27], albeit after DNAse treatment with a TURBO DNA-free kit, a phenol/chloroform/IAA step was included, followed by two cycles of chloroform/IAA extractions. For precipitation, a 1/20th volume of 5 M NaCl and two volumes of ethanol were added, and then the solution was incubated for 20 min at −20 °C. RNA purification was carried out with Qiagen spin columns, washing with Qiagen washing solutions, and finally elution with 50 microliters of RNase-free water. The quantity and purity of the total RNA samples were assessed by ultraviolet spectroscopy using a DS-11 spectrophotometer (DeNovix, Inc., Wilmington, North Carolina, USA).

### 3.13. Relative RT-qPCR for the Estimation of Biofilm-Associated Gene Expression Following EG Exposure

One microgram of total RNA was reverse-transcribed using modified MMLV RTase, a random hexamer primer included in the qPCRBIO cDNA Synthesis Kit (PCR Biosystems, Ltd., London, England). The qPCR was performed using a Biorad CT030008 system (Biorad,) with SSo SyGreen Mix (Biorad Ltd., London, England). The total reaction volume was 20 µL; one µL of each cDNA sample was used, and the final primer concentration was 400 nM for both the forward and reverse primers, in accordance with the manufacturer’s instructions. The cycling conditions were as follows: 30 s of initial denaturation at 95 °C, 40 cycles consisting of 30 s at 95 °C and 30 s at 60 °C, and a final melting curve program. The primer sequences were as previously published [13].

Amplifications using total RNA that was not reverse-transcribed were performed to check for genomic DNA contamination, and no-template controls were included. The comparative ∆∆C_T_ Livak method for qPCR data was applied as a standard procedure in the analysis of the relative gene expression data. The *C*_T_ (cycle threshold) values obtained from the experimental RNA samples were normalized to the reference gene 16S rRNA, and the difference in the ∆*C*_T_ values (∆∆*C*_T_) between the samples of interest and the control samples was calculated [28].

### 3.14. Statistical Analysis

The statistical evaluation of the data obtained from the colorimetric, profilometric, and pH measurements was performed using the Statistical Package for the Social Sciences software, version 23.0 for Windows (IBM Corp., Armonk, NY, USA). The differences between the untreated control and treatment groups were assessed by applying a one-way analysis of variance, followed by a post hoc least-significant difference test for the comparison of multiple means. The data are represented as the mean ± standard error. The statistical significance was set at a *p* value of less than 0.05. In the gene expression experiments, three independent experiments with two technical tests were conducted for each treatment (*n* = 6). The statistical analysis was performed using a one-way analysis of variance, followed by the Tukey-Kramer test, at a significance level of 0.05. The figures display the mean and standard deviations.

## 4. Conclusions

The accumulation of biofilm produced by *Streptococcus mutans* bacteria on hard tooth tissues remains one of the most prevalent oral diseases, so that the development of new antibiofilm agents is of critical importance. Our results show that C8-OG significantly diminished biofilm formation by *S. mutans* exposed to it on solid surfaces and suppressed acidogenicity in a dose-dependent manner, compared to unexposed bacteria (*p* < 0.05). The C8-OG concentration of 100.24 µM inhibited the *S. mutans* biofilm development on solid surfaces by 100% and prevented a decrease in pH levels by 99%. The highest concentration of C8-OG (100.24 µM) reduced biofilm formation on polystyrene and glass surfaces by 100% and prevented a decrease in pH levels by 99%. Due to the capability of C8-OG to inhibit biofilm formation and acidogenicity, it might be utilized as an anticaries agent for oral formulations that aim to reduce the prevalence of dental caries.

In the current study, we have investigated the effects of C8-OG treatment on five important genes involved in biofilm production by *S. mutans*. The results show that biofilm-producing bacteria treated with C8-OG exhibited notable changes in gene expression for four of the genes under study (*gbpB*, *gtfc*, *gtfD*, and *atpD*) and a slight decrease in the expression of the *gtfB* gene. However, C8-OG treatments did not produce significant expression change compared to the control for the planktonic cells, although there was a significant increase for the *atpD* gene only. In the future, more studies will be conducted to follow the antibiofilm effect after treatment with other modified gallate derivatives.

## Figures and Tables

**Table 1:** Antibacterial activity of octyl gallate (stock solution 100 mg/mL dissolved in pure dimethyl sulfoxide (DMSO)), erythromycin (positive control, stock solution 10 mg/mL dissolved in DMSO), and DMSO (solvent).

Compound	MIC
Octyl gallate	97 µg/mL (343.5 µM)
Erythromycin	4.8 µg/mL (6.54 µM)
DMSO	25% (*v/v*)

**Table 2:** Effects of C8-OG on the pH levels of the *S. mutans* biofilm growth medium after 24 h of incubation in the presence of 1% sucrose.

Experimental Group	pH
Blank	7.37 ± 0.02 *
Control	4.23 ± 0.01
DMSO (0.57%)	4.22 ± 0.01
C8-OG (97.4 µM)	6.27 ± 0.38 *
C8-OG (98.11 µM)	6.83 ± 0.23 *
C8-OG (98.82 µM)	7.19 ± 0.04 *
C8-OG (99.53 µM)	7.23 ± 0.03 *
C8-OG (100.24 µM)	7.28 ± 0.03 *

The data represent the mean ± standard error from three separate experiments (*n* = 3–9). * *p* < 0.05 versus the control group.
